# Lectures on the Diagnosis and Treatment of Diseases of the Lungs

**Published:** 1840-10-17

**Authors:** W. W. Gerhard


### MEDICAL EXAMINER.

DEVOTED TO MEDICINE, SURGERY, AND THE COLLATERAL SCIENCES.

No. 42.] PHILADELPHIA, SATURDAY, OCTOBER 17, 1840. [Vol. III.

### LECTURES ON THE DIAGNOSIS AND TREATMENT OF DISEASES OF THE LUNGS.

BY W. W. GERHARD, M. D.

### LECTURE XI.

#### PNEUMONIA.

In my last lecture I finished the subject of diseases of the mucous membranes lining the bronchial tubes. As I had previously described the inflammation of the investing membrane, it now only remains for me to give an account of the affections of the parenchyma of the lungs. It was necessary to treat of the diseases of the membranes first, because the parenchyma is very rarely, if ever, diseased, without the inflammation extending to them, for the tendency of disease of the lungs is to produce inflammation of the mucous and serous membranes connected with them. What, then, is the parenchyma? To answer this question, it will be necessary to run over the anatomical structure of these viscera. The bronchi continue to divide and subdivide, the ramifications becoming smaller and smaller after each division, and they are arranged in lobules, the vesicles of each communicating with one another, but not with those of the adjoining lobules; and each lobule receives a bronchial tube, which ramifies within it, and is distributed to the vesicles in the cellular tissue, which invests and unites them together. The parenchyma may, then, be said to consist of the air-vesicles, the blood-vessels surrounding them, and the cellular tissue; or the term may be extended further, so as to include the ramifications of the tubes within the lobules, yet not the tubes which lead to them. The latter definition answers better in a pathological view, inasmuch as the smaller tubes are always involved in diseases of the portion of the parenchyma through which they pass. The term parenchyma being then understood to include these finer tubes, we will designate the disease as bronchitis when the inflammation attacks the large bronchial tubes, extending no further than the tubes which lead to the lobules: pneumonia, when it extends to the smaller tubes within the lobules, and the air-cells of the part affected.

Pneumonia, which is an inflammation of the parenchyma of the lungs, may commence in two ways,—either as a bronchitis, the inflammation in this case extending to the smaller tubes and air-vesicles; or it may originate in the vesicular structure, and subsequently involve the larger tubes, just as dysentery may commence in the form of diarrhœa, and pass into dysenteric inflammation, or originate in the latter form, and present the symptoms of dysentery from the first. When the bronchial tubes only are inflamed, as soon as a secretion takes place it is removed from the body, and the inflammation is partially relieved, and the disease rarely does much harm; but when the lobules are inflamed, the exit is closed, and the fluid accumulates in the lung, thus increasing the congestion, and impeding the respiration, but not relieving the inflammation by a natural depletion. This fluid consists at first of a bloody serum, and is often of a reddish colour. It afterwards passes through the stages of lymph and pus. In hæmorrhagia, the blood contained in the cellular tissue is arterial in its character; in apoplexy, it is venous, and in inflammation it partakes in a measure of the nature of both. The lung at this stage of the disease, yields readily to pressure with the finger, and the fluid can be expressed from it. In post-mortem examinations this appearance may be confounded with engorgement produced after death, in a dependent portion of the viscus; and there is frequently some difficulty in making the distinction between the two,—but the redness of inflammation is always brighter, and the softening of the tissue is more decided. Still the two conditions are not very dissimilar, for the congestion, if it occur during life, may readily pass into inflammation.

Pneumonia passes through several stages between its commencement, which I have described, and termination; and its symptoms, in accordance with the changes of structure, are divided into four stages. The first is characterized by engorgement of the tissue; the second by induration, which has received several names, as hardening, red softening, hepatization. It is called hardening, on account of the increased consistency which is perceived when slightly pressed; softening, on account of the facility with which it is broken, if the pressure be increased; hepatization, from its resemblance to the tissue of the liver. The vesicles of the lung being deprived of air, and engorged with blood, resemble the acini of the liver, their colour being thus changed to a brownish red. In the case of children, this resemblance is so clear, that I have known a bystander to mistake a piece of lung for liver, although both tissues were before him. A small piece of lung in this stage of the disease will sink in water, although a large mass of it may float on account of some portion of it containing air in its cells; whereas, in the first stage, the whole of the tissue is lighter than water. The bronchial tubes are red, and filled with a fluid containing a large portion of lymph, which in many cases closes the smaller tubes, thus reducing the lung to an uniformly solid mass. If the lung be torn, or even if simply cut, it presents an irregular granulated appearance, which arises from the vesicles being separately hardened and enlarged, but still retaining their individual form. They therefore project above the level of the adjoining cellular tissue.

In the third stage the lung remains indurated, but assumes a yellowish colour. In this stage the lung contains a considerable quantity of pus, diffused through the cellular tissue, and deposited in the vesicles. The tissue loses its granular appearance, and becomes more smooth and polished, the vesicular structure having been completely obliterated. It yields readily to pressure, and breaks under the finger, affording a puriform liquid, which at first consists of a mixture of pus and blood globules floating in serum, and afterwards of pure pus. By placing the diseased lung under a stream of water, the parenchyma may be completely removed so as to leave nothing but the bronchial tubes. The bronchial mucous membrane is not so red in this as in the second stage, and the tubes contain purulent liquid.

We may admit a fourth stage, in which the parenchyma is softened down and removed by expectoration, and an abscess remains, resembling an abscess in the other tissues of the body; a pus-secreting membrane is formed, and pus is thrown out, which becomes less and less in quantity until cicatrization takes place, and a cure is effected. This stage of the disease is rarely met with; but when it does occur, the patient usually recovers,—which termination you would not expect, as an abscess in the lungs appears to be a lesion of great gravity. However, if the patient has strength enough to go through the first three stages of the disease, he will generally survive the fourth, though he may require the aid of artificial stimulants. The symptoms are generally somewhat relieved by the formation of an abscess, as the inflammation is thus circumscribed in its locality. If, however, instead of there being a circumscribed abscess, the pus be diffused through the lung, a fatal termination will generally take place.

The physical signs of pneumonia, like the lesions, occur in a regular series. The signs in the first stage are obscure, but in the second they become very plain; hence they are looked upon as the pathognomic signs of the disease. In the first stage, the lung is infiltrated with a thin liquid; this produces a sort of rustling respiration, and not unfrequently the respiration at the time is rude; that is, the vesicular murmur loses its natural softness and fulness, and the air rushes abruptly into the cells. Subsequently we meet with another sign, which is said to be pathognomic of the first stage. This is the crepitant rhonchus. It is indeed pathognomonic when it does exist, but it is not present in all cases; for when the inflammation is seated near the centre of the lung, the engorged vesicles cannot dilate; as this rhonchus is produced by the expansion of the diseased vesicles, of course it cannot be heard. Besides, the healthy tissue, which is to be found between the ear and the diseased lung, gives rise to a healthy vesicular respiration, and prevents the crepitus from being heard after it is formed in the inflamed portion. But when the seat of the inflammation is near the surface, it always occurs. There is also slight dulness on percussion, which is caused by the secreted liquid partially displacing the air in the tubes. The dulness is of course not considerable, for the air is not completely expelled from the diseased portion.

The signs of the second stage are more strictly pathognomonic of the disease. In this stage the tissue of the lung is completely altered, and this alteration is attended with corresponding physical signs. On percussion we find complete flatness, as the cells are filled with fluid, and no air whatever is contained within them. Auscultation gives us, 1st, a bronchial respiration, which is more marked in the second stage of pneumonia than in any other affection of the lungs, as the tissue is perfectly consolidated without any obliteration of the tubes. It is that variety of bronchial respiration, which, on account of its loudness, has been denominated tubal; it is most distinctly heard at the root of the lung, where the tubes are of the greatest calibre. Bronchial resonance of the voice, or bronchophony, is also heard, and in fact it always coexists with the bronchial respiration. If the patient breathes rapidly, the crepitant rhonchus is also heard in many cases co-existing with the bronchial respiration; this arises from a portion of the lung remaining in the first stage of inflammation. It is then heard in trains, like the crackling of wet powder, in the tissue which has not been indurated, and which surrounds the solidified portion. This state of things is very frequently met with. These signs are present in all cases except when the patient breathes too feebly to impel the air through the tubes, when, of course, they are not heard; but as they are so constantly met with they are usually described as the pathognomonic signs of pneumonia. The patient should always be directed to cough when you suspect that he is in the second stage of pneumonia; and you will then find that the bronchial respiration is made much more distinct, and the air is driven so suddenly into the smaller tubes during the following inspiration, that a very characteristic crepitus is produced either in the same spot as the bronchial respiration, or very near it, for the lung can never be completely solidified.

The signs of the third stage are not so characteristic of the disease; but, if you have followed it through the previous stages, you cannot be at fault, nor can you, if the signs of the first and second, or of the three stages be present at the same time. But if you see the patient for the first time in the third stage, you may, by relying on the general symptoms, mistake the disease for an affection of the brain, which it sometimes much resembles. The signs are, 1st, those connected with percussion, which is perfectly flat, as the lung remains solid, and very little air is contained in the tubes. The results given by auscultation are obscure, as the current of air has by this time been diverted from the diseased lung, just as the blood is diverted from a gangrenous limb, and therefore little or no sound is heard. The respiration, when heard, is feebly bronchial; a mucous rhonchus is also present. We have, then, as signs of the third stage, flatness on percussion, feeble bronchial respiration, and mucous rhonchus. The resonance of the voice is proportional to the respiration, and is of course feeble. These signs are all very obscure, and therefore you must expect to be foiled when called to a patient in this stage of the disease. There may still be heard in very strong inspirations a decided crepitous rhonchus; but this is rather owing to a portion of the lung which remains still in the first or second stage, and admits the air in very strong inspirations.

The signs of the fourth stage, or that of abscess, are the usual signs of formation of a cavity, viz. at first a mucous rhonchus becoming more loose and large, until at last a well developed gurgling is heard, produced by the passage of air through the pus contained in the cavity. The following table will give you a condensed view of the physical signs connected with the different stages of the disease:

| | | |
|---|---|---|
| First stage, or engorgement. | Rude or harsh respiration; crepitant rhonchus. | Percussion clear, or nearly so. |
| Second, or hepatization. | Bronchial respiration; bronchophony; crepitant rhonchus around it. | Percussion flat, or very dull. |
| Third, or purulent infiltration. | Bronchial respiration in large tubes feeble, or absent elsewhere; mucous and subcrepitant rhonchus; bronchophony imperfect. | Percussion flat. |
| Fourth stage, or abscess. | Cavernous respiration gurgling. | Percussion flat. |

In practice, several of these stages may coexist in the same lung; but the signs of each may be recognised without difficulty, and the proportionate extent marked out with tolerable precision.

When the disease terminates by recovery, it gradually retraces its steps until it returns to a healthy state. The signs connected with this return to health are called the *signs of return*, or of recovery: their regularity depends upon the stage which the disease had previously reached. If the disease advance no further than the second stage, it will regularly return to the first. When first the crepitant rhonchus of return is heard, it is looser or more moist than the true crepitant; this gradually subsides, and the vesicular respiration re-appears, but remains for a long time much more feeble than it was previously to the attack. The bronchial respiration and dull percussion do not suddenly cease, but remain in some degree for a considerable time after the cessation of most of the symptoms of the disease. This depends upon the consolidation of the lung, and the difficulty with which the tissue returns to its vesicular expansive condition.

When, however, the disease has reached the third stage, this series of changes does not occur. The mucous rhonchus is the first sign observed, as a large quantity of fluid is poured into the tubes. The crepitant rhonchus of return is not heard, as no air passes through the smaller tubes. This fluid, which is produced in the bronchi, consists of mucous and purulent matter, resulting from the breaking down of the diseased tissue, and the secretion from the tubes passing through the inflamed mass, which contributes very much to the relief of the disease. This secretion gradually assumes more and more of the mucous character until it becomes perfectly natural.

The return from the fourth stage is marked by the secretion of pus becoming less and less, and at last disappearing with the cicatrization of the parts involved in the abscess, while the secretion becomes entirely mucous in its character.

These stages belong to pneumonia of a perfectly frank character; they are, however, liable to be modified by various circumstances which are necessarily attendant upon the disease. There are some lesions always found in pneumonia,—that is, inflammation of the pleura, and of the bronchial mucous membrane. The pleurisy is at first dry, and merely produces slight pain and a feeble sound of respiration. When the pleurisy is slight, the affection is simply called pneumonia; when the pleurisy is severe, and attended with a large effusion, it is called *pleuro-pneumonia;* and when the pleurisy is considerable, with very slight inflammation of the parenchyma, it is merely termed *pleurisy*. When the pleuritic effusion is considerable, the signs of one or the other affection predominate according to the relative stage of each disorder; the pneumonia is apt to decline sooner than the pleurisy, which may remain for an indefinite period after the cessation of the inflammation of the substance of the lung.

The bronchitis which attends pneumonia may be confined to the tubes which lead to the lobules, or it may extend throughout the bronchial tree; that which is confined to the inflamed portion of the lung is always present to a greater or less degree; but the general bronchitis is extremely variable, and generally takes place under two different circumstances. In one the bronchitis occurs as an ordinary catarrh, and the pneumonia occurs afterwards during its progress. In the other the bronchial affection comes on late in the disease, and generally in the third stage of it, when the purulent secretion is copious, and passes into the bronchial tubes.

Having given the physical signs of frank pneumonia, we shall now proceed to consider the functional signs of this affection. These are of three kinds—*local*, *secondary*, and *general.* The local comprise cough, expectoration, frequency and mode of performance of the respiration, and the pain produced by the act of breathing. By *secondary* signs we mean the affections of the brain, alimentary canal, the assistant chylopoietic viscera, &c. The general signs are those which are common to all inflammatory affections, as the condition of the circulation, &c.

*Local signs*, cough, &c. The cough is usually at first the ordinary cough of acute bronchitis, which is either hoarse, or a loose mucous cough. In this case the bronchitis is the predominant affection; as soon, however, as the parenchyma becomes seriously affected, the cough changes its character, assuming the form which is called pneumonic. The *pneumonic* cough is short and suppressed, which results partly from the pain felt during the act of coughing, and partly from the impossibility of inflating the lungs completely; hence the force of the column of air, which is expired during the act of coughing, is not sufficient to cause a loud and distinct sound. The The pneumonic cough begins from the first, if the disease attack the parenchyma and pleura before passing through ordinary bronchitis. The cough sometimes exhibits this character from the first. In some cases of pneumonia the cough is wanting throughout the course of the disease, which is then said to be *latent;* in such cases the patient is generally aged, or the pneumonia succeeds another affection. As the disease proceeds, secretion takes place, and the cough again becomes loose; when an abscess is formed, it becomes exceedingly loose and rattling.

The frequency of *respiration* is increased in pneumonia, and the degree of increase is a tolerably exact indication of the extent of the affection. This frequency of respiration arises from the diseased lung being rendered unfit for the performance of its functions, so that a smaller portion of the blood is exposed at once to the action of the air, and a smaller quantity of air is inhaled during an inspiration. Therefore it must be changed more frequently. Besides, the inflation of the healthy portion is less complete than natural, because the motion of the lungs is suspended, and the action of the respiratory muscles is less complete. Where only one lung is slightly diseased, the frequency of the respiration is but very little increased. If the disease embraces the whole of one lobe of the lung, it is increased to forty or fifty a minute; and when both lungs are involved, the respiration will be as frequent as fifty or sixty in the minute. Should it be more frequent, the extent of the mischief is very great. It must be evident, then, that this sign is important for the prognosis of the disease. The mode of performing respiration differs from that observed in the healthy state. The patient breathes irregularly; the respiration is usually high, and is performed chiefly by one side of the chest. At first it is not strictly abdominal; but after pneumonia has continued for a time this character is developed, and then the ribs remain nearly motionless.

The *pain* is very variable, and is proportioned to the inflammation of the pleura. When the inflammation is situated near the surface of the lung, the pleura is necessarily much involved, and the pain is consequently acute; but when it is deep-seated, there is, generally speaking, little or no pain. In the old and feeble the pain is scarcely felt, whatever be the portion involved. Therefore, as in many cases it is wanting, and as, when present, it does not indicate the extent of the pulmonary inflammation, it is a sign of very little importance.

The *expectoration*, in the commencement of pneumonia, consists of mucus, such as is observed in ordinary bronchitis, and differs but little from the healthy secretion. As the disease is developed, it becomes viscid and transparent, and in some cases is of a rusty colour; the viscidity and transparency are the characteristic properties of the *pneumonic sputa.* It is sometimes so viscid that it will not flow from the vessel containing it, although the latter be turned bottom upwards. It is small in quantity, generally from one to four ounces in twenty-four hours; its becoming more abundant is a sign that the disease is retrograding, sometimes it is mixed with yellow sputa from some other portion of the lung or tubes. As the disease passes from the second into the third stage, we observe an admixture of pus, and when it declines the sputa become thinner and more mucous in their character. If an abscess form, the sputa is decidedly purulent, and a large quantity is either suddenly discharged or expectorated in a very short time. This is, to some extent, the case, when the third stage is so far advanced that a considerable portion of the lung is softened into a pulp, even if there be no large cavity.

The *secondary* signs may be divided into those connected with the lungs, and those dependent upon other organs.

*Affections of the lungs.*—Bronchitis and pleurisy almost always attend pneumonia, but their severity varies exceedingly. Tubercles are sometimes formed in the lung during the course or in the decline of pneumonia, which, though it is not probably the sole cause of their formation, in many cases hastens their development. Their formation, of course, increases very much the gravity of the prognosis. Emphysema is sometimes produced during an attack of pneumonia, principally in children. This lesion is not so important when it is an affection owing to pneumonia, as when it has existed previously to the occurrence of the latter affection, in which case, by increasing the dyspnœa, it renders the prognosis more unfavourable.

The heart is very often secondarily affected in this disease, sometimes from the general diffusion of the inflammatory action, and sometimes from the imperfect performance of the function of respiration, the blood becomes congested in the right ventricle, and in some cases a coagulum is formed in consequence of the imperfect circulation, and of the highly fibrinous state of the blood. But often, in addition to this, we find inflammation of the lining membrane of the left ventricle, which is more frequently affected in this manner than the right, in consequence of the general law, than the arterial system is more subject to inflammation than the venous. This occurs in a large proportion of the severe cases of pneumonia. This affection of the heart varies in intensity—sometimes the membrane is merely reddened, sometimes it is opaque and thickened, partly by the deposition of lymph, and occasionally it is ulcerated; the ulceration is generally seated at the valves.

The *brain* is very often affected in pneumonia, and when the inflammation of this organ occurs, it is attended by delirium, such as takes place in common arachnitis. The medullary cerebral substance is not often the seat of the inflammation, which in almost all cases is confined to the membranes, and to the cortical substance. Dr. Louis says that one-sixth of the cases of pneumonia which he saw, were complicated with an affection of the brain; like the inflammations of the heart, it occurs most frequently in the very severe cases of pneumonia. If the cerebral symptoms should be severe, the primary affection is generally masked by the secondary, which often gives rise to an error in diagnosis, as the signs of arachnitis are very evident while the functional signs of pneumonia are observed, and, therefore, liable to be overlooked. This complication adds very much to the gravity of the prognosis; and unless active treatment be resorted to at the commencement of the attack, it is very apt to prove fatal.

The liver is sometimes involved in pneumonia, but the frequency of this complication varies at different seasons, and in different localities, being much more common at our southern Atlantic coast than it is at the north. This inflammation of the liver is distinguished by some authors from bilious pneumonia, although it closely resembles it, and as it seems to me, differs only in the bilious pneumonia described by Stoll, being an epidemic disease. Its signs are jaundice, pain in the side and shoulder, and cerebral symptoms, such as stupor and somnolency, which are dependent upon it. Bilious pneumonia, though in some years common amongst us, is now very rare. It differs from pneumonia, in which the affection of the liver is a mere secondary complication, by the liver being attacked, in bilious pneumonia, simultaneously with the lung.

The right lung is the one which is always most inflamed in pneumonia complicated with the inflammation of the liver, and the extension of the inflammation from the lung to the liver, in the simultaneous attacks of the two organs, shows that there must have been previously a disorder of the liver, which favoured, at least, the extension of the disease, hence the affection is so frequent in warm climates and miasmatic situations. This complication certainly adds much to the difficulty of diagnosis without the physical signs, especially as the cerebral symptoms are generally so well marked as to suppress, in a great degree, the cough.

Inflammation of the stomach and bowels, of the œsophagus and pharynx, have all been observed in pneumonia, and also inflammation of the kidneys; but these complications are not more common in this than in other inflammatory diseases. They may be known by their proper local signs, and I shall therefore not detain you with a minute account of them.

*General signs—Capillary circulation.*—A sign which may be called general, although confined to very narrow limits, is the appearance of the face, for this depends upon the capillary circulation. In acute cases we meet with a circumscribed flush of a circular form, and which is sometimes confined to one cheek, sometimes found in both. When one cheek only is affected in this manner, it is more frequently, though not invariably, that which corresponds with the diseased lung. In some cases the whole face is flushed, the colour varying from a light to a deep red; sometimes it is of a bluish colour. The whole countenance is generally changed. These various tints depend upon the greater or less obstruction of the circulation of the blood through the heart and lungs, they are darker when the difficulty of the circulation is greater, and often become bluish about the lips and nostrils, while the rest of the face is pale, if a coagulum should form in the heart. Dilatation of the nostrils in each inspiration, is another symptom; this depends upon the dyspnœa, and its extent is in proportion to the latter.

*General circulation.*—The disease makes its appearance in the following manner: The patient is first seized with a chill; this lasts half an hour or more, and sometimes two or three hours, and when it goes off is succeeded by a fever, which continues during the whole twenty-four hours, but usually increases at night, and is rarely attended with extreme heat of skin. The pulse is full, hard, and developed at the commencement of the disease; in the latter stages it is frequently feeble. It is very generally from one hundred to one hundred and twenty, and rarely becomes more frequent, except in the terminating stage of the disease. It is in most cases a good measure of the intensity of the inflammation, and a correct indication of the propriety of blood-letting; but the pulse is sometimes contracted, and at the same time the inflammation is violent; if bleeding be practised it rises and becomes softer. A careful bleeding, if the general symptoms be inflammatory, is the best guide in this matter.

The alteration of the strength is another sign which is connected with inflammatory diseases, in general, the degree of diminution depending upon the importance of the part affected, and the extent to which the inflammation proceeds. Thus, a patient with pleurisy, will continue to walk about until the effusion causes so much dyspnœa that he is compelled to keep his bed; whereas a slight pneumonia, with scarcely any local signs, will enfeeble him so much that he will be unable to sit up.

Although the physical signs are the most important in the diagnosis, as they indicate the extent as well as the nature of the affection; yet there are certain rational signs, which, taken together, may be considered as pathognomonic, namely, the expectoration, flush, and dyspnœa: these are, however, often obscure at the commencement of the attack. The physical signs are often only required to ascertain the extent of the disease, as its character is rendered sufficiently apparent from the rational signs of sthenic pneumonia.

*Prognosis.*—The prognosis is very variable in all diseases of this kind, as it often depends upon circumstances unconnected with the disorder itself. In ordinary frank pneumonia the prognosis is favourable where other things are not unfavourable; that is, where it attacks a person previously in good health, and the treatment is commenced early, for this modifies the disease very much, when begun at an early period; but after it has continued a few days, the prognosis is very little affected by it. When it is complicated with an affection of the brain or liver, the prognosis is more unfavourable.

*Duration.*—A frank pneumonia without treatment usually lasts from ten to twenty days, but if it has reached the third stage, it will last much longer. If it has continued a few days before the commencement of the treatment, it rarely ends before the tenth day. If you treat it from the first, you may frequently produce a partial jugulation of the disease, and shorten somewhat its duration. The observations made at Paris coincide in this respect with the experience of Dr. Jackson, of Boston, and the results obtained in this city. When the disorder terminates fatally, death usually occurs early in the third stage, or just in the passage from the second to the third stage. This stage is reached in different periods, sometimes in three or four days, but generally about the beginning of the second week.

